# Comparison of ultrasound-guided serratus anterior plane block and thoracic paravertebral block in postoperative analgesia and inflammation control in patients undergoing upper abdominal surgery

**DOI:** 10.12669/pjms.39.1.6849

**Published:** 2023

**Authors:** Zhiming Cai, Ruiqun Xie, Ting Xu, Changlu Huang

**Affiliations:** 1 Zhiming Cai, Department of Anesthesiology and Perioperative Medicine, 900 Hospital of the Joint Logistic Support Force, Fuzhou, Fujian Province, 350025 P.R. China; 2 Ruiqun Xie, Department of Anesthesiology and Perioperative Medicine, 900 Hospital of the Joint Logistic Support Force, Fuzhou, Fujian Province, 350025 P.R. China; 3 Ting Xu, Department of Anesthesiology and Perioperative Medicine, 900 Hospital of the Joint Logistic Support Force, Fuzhou, Fujian Province, 350025 P.R. China; 4 Changlu Huang, Department of Anesthesiology and Perioperative Medicine, 900 Hospital of the Joint Logistic Support Force, Fuzhou, Fujian Province, 350025 P.R. China

**Keywords:** Ultrasound-guided serratus anterior plane block, Thoracic paravertebral block, Upper abdominal surgery, Analgesia, Inflammatory response

## Abstract

**Objective:**

To compare the effects of ultrasound-guided serratus anterior plane block (SAPB) and thoracic paravertebral block (TPVB) on postoperative analgesia and inflammation control in patients undergoing upper abdominal surgery.

**Methods:**

This is a retrospective observational study. The records of patients who underwent upper abdominal surgery in our hospital from June 2019 to January 2021 were selected and retrospectively divided into two groups based on the analgesia method. Fifty-nine patients received ultrasound-guided SAPB analgesia (SAPB-group) and 55 patients received ultrasound-guided TPVB analgesia (TPVB-Group). Patients were matched for age, gender and body-mass index (BMI). The visual analogue scale (VAS) scores of pain at two hours(T1), six hours (T2), 12 hours (T3), 24 hours (T4) and 48 hours (T5) after the operation were compared between the two groups. The levels of interleukin-6 (IL-6), interleukin-10(IL-10) and tumor necrosis factor-α (TNF-α) at the completion of surgery (T0) and T4 were compared between the two groups.

**Results:**

The duration of block in SAPB-group was higher than that in TPVB-group (*P*<0.05). VAS scores of SAPB-groups were significantly lower than those of TPVB-group at all-time points (*P*<0.05) except at rest 48 hour after the procedure. The levels of IL-6, IL-10 and TNF-α at 24 hours after the operation in both groups were significantly higher than immediately at the end of the operation (*P*<0.05). Levels of IL-6 and TNF-α 24 hours after the operation were significantly lower in the SAPB-group than in the TPVB-group (*P*<0.05), while the levels of IL-10 24 hours after the operation were significantly higher in the SAPB-group (*P*<0.05).

**Conclusions:**

SAPB block under ultrasound guidance for patients undergoing upper abdominal surgery has good anesthetic and analgesic effect and can significantly improve the level of postoperative inflammation.

## INTRODUCTION

Upper abdominal surgery is often used in cases of radical mastectomy for breast cancer and radical resection of lung cancer and is accompanied by intraoperative and postoperative pain that may affect the prognosis of patients. Preoperative use of high-dose opioids can inhibit hemodynamic disturbance caused by intraoperative stimulation.^1^,^2^ However, clinical studies show that high-dose opioids may negatively affect postoperative recovery of patients undergoing upper abdominal surgery due to frequent adverse effects, such as respiratory depression.^3^

In recent years, with the development of ultrasound and regional anesthesia techniques, ultrasound-guided regional block became increasingly popular in clinical setting.^4^ Ultrasound guidance allows to visualize needle insertion in real time, and therefore prevent possible discomfort due to current stimulation of motor nerve and improve the safety of peripheral nerve block.^5^ Ultrasound-guided TPVB and SAPB are two most common analgesic methods that are simple, associated with high safety and good analgesic effect, and are widely used in radical operations such as radical mastectomy, radical resection of lung cancer, and other upper abdominal operations.^6^

TPVB has achieved good results in thoracotomy in elderly patients and can better maintain their perioperative hemodynamic level.^7^ SAPB is a new chest wall block technology that was developed in recent years and has significant advantages in postoperative analgesia after thoracoscopic surgery.^8^ The main objective of this study was to compare the efficiency of ultrasound-guided SAPB and TPVB for postoperative analgesia and inflammation control in patients undergoing upper abdominal surgery.

## METHODS

A retrospective observational study was conducted. Medical records of 114 patients who underwent upper abdominal surgery in our hospital from June 2019 to January 2021 were retrospectively selected, including 66 males and 48 females. Of them, 59 patients received ultrasound-guided SAPB analgesia, and 55 patients received ultrasound-guided TPVB analgesia. Patients were matched for age, gender and BMI. The general data were compared between the groups (*P* > 0.05), which suggested comparability. The study was conducted and reported based on the STROBE guidelines.^9^ The study was approved by the ethics committee of our hospital (No. 2021024; Date: Jan. 10th, 2021) and informed consent was taken from all participants.

### Inclusion criteria:

- Patients classified as grade I-II based on the guidelines of the American Society of anesthesiologists.^10^
- Age: 29~75 years
- All patients underwent upper abdominal surgery
- All patients were treated with upper abdominal surgery, including open surgery for stomach, gallbladder and liver diseases.

### Exclusion criteria:

- Past history of allergy to ropivacaine anesthetics and opioid analgesics
- Patients with severe cardiovascular disease, malignant tumor of blood system, and chronic pain
- Patients with abnormal coagulation function
- Infection at the puncture site
- Patients with incomplete clinical data.

### Anesthesia induction:

Patients were fasting within eight hours before the operation and stopped drinking two hours before the operation. Routine preoperative preparation included electrocardiogram (ECG), heart rate (HR), oxygen saturation (SpO_2_), and bispectral index (BIS) that were monitored after entering the room. Oxygen was administered by mask, and peripheral arterial and venous access was established. Both groups underwent ultrasound-guided regional nerve block before induction of anesthesia. For the induction of anesthesia, 1μg/kg dexmedetomidine (Jiangsu Hengrui Pharmaceutical Co., Ltd., approval no. 20090248), 0.4μg/kg sufentanil (Yichang Humanwell Pharmaceutical Co., Ltd., approval no. 20120814), 2.5mg/kg propofol (Guangdong Jiabo Pharmaceutical Co., Ltd., approval no. 20051842) and 0.75mg/kg rocuronium (Jiangsu Nhwa Pharmaceutical Co., Ltd., approval no. 20143315) were intravenously injected. A double-lumen bronchial catheter was inserted, and mechanical ventilation was performed after the fiberoptic bronchoscope was aligned

### Anesthesia maintenance:

Anesthesia was maintained by 30-70 ug/Kg/min of propofol and 0.2-0.4 μg / (kg· minute) remifentanil, and 0.1-0.2 mg/kg atracurium CIS benzenesulfonate (Hainan Huanglong Pharmaceutical Co., Ltd., approval no. 20183357) to keep the heart rate and mean arterial pressure within 20% of the baseline, and BIS at 40 ~ 60. Thirty minutes before the end of the operation, we stopped pump injection of atracurium CIS benzenesulfonate and sufentanil 1.5 μg/kg was given. After that, extubation was performed when the patients became fully awake.

After the operation, all patients received patient-controlled intravenous analgesia (PICA) via analgesic pump, and 100 ml of sufentanil was prepared with 100 UG normal saline. The protocol of PICA was set as follows: background infusion of 2ml/hour, 2ml bolus and locking time of 10 minutes. Visual analogue scale (VAS) was used to quantitatively assess the pain in quiet and cough state of the patients.^11^ VAS represents different pain levels on a scale of 0-10, with 0 being no pain and 10 being severe pain. If the patient’s VAS pain at rest was over three points or cough VAS score exceeded six points, PCIA and intravenous infusion of 5μg sufentanil were continued to relieve the pain. PCIA was administered until 48 hours after the operation.

### Analgesic methods:

For the SAPB block under ultrasound guidance, the patient was placed in the supine position, with the upper arm abducted and the elbow flexed. The ultrasound probe was placed at the right axillary midline of the patient, at the 4th and 5th ribs, to find the images of superficial latissimus dorsi and deep anterior serratus. The 22G block needle was advanced until it crossed the latissimus dosrsi to the deep surface of anterior serratus. After confirming that there is no blood return and no air, 20 ml dose of 0.33% ropivacaine (Hebei Yipin Pharmaceutical Co., Ltd; approval no. 20113463) was injected slowly, and the diffusion range of local anesthetic drugs between fascia’s was observed.

For the TPVB block under ultrasound guidance, the patient was placed in the lateral decubitus position, the position of T5 spinous process was confirmed, and the ultrasonic probe was placed at the position where T5 spinous process is perpendicular to the posterior median line, and the inner end of the probe is at the posterior median line. The position of T5 spinous process and T6 transverse process could be clearly observed, and the position of the paravertebral space of the probe could be moved laterally (the space surrounded by transverse process, superior costal transverse process ligament and pleura). After fixing the probe, the plane medial technique was used to enter the needle from the back of the probe. When the needle tip reached the T5 paravertebral space, it was drawn back and confirmed that there was no blood and air return. Ropivacaine was then slowly injected, and the diffusion process of sub parietal local anesthesia in the paravertebral space was monitored. The procedure was performed using portable Sonosite EDGE II Ultrasound ultrasonic instrument with the following parameters of the probe: 6~13Hz high-frequency linear array probe. Acupuncture was used for all patients to confirm the sensory block level 30 minutes after the end of block. After the effect was confirmed, bronchial intubation and further intravenous anesthesia were implemented.

### Basic characteristics and indexes:

The following data and indexes were collected: onset time, duration of block and anesthesia plane; pain VAS scores at rest and cough at two hours (T1), six hours (T2), 12 hours (T3), 24 hours (T4) and 48 hours (T5) after the operation; serum levels of IL-6, IL-10 and TNF-α at the end of operation (T0) and T5 after operation were detected from 4ml of fasting venous blood. Blood was collected in non-anticoagulant tubes, centrifuged and separated from the serum (Rate: 3000r/minutes, duration: 10minutes). Enzyme linked immunosorbent assays (ELISA) was used to detect interleukin-6 (IL-6), interleukin-10 (IL-10) and tumor necrosis factor α (TNF-α) using a small Vipas automatic fluorescent enzyme labeling instrument. The reagent and kit were provided by Shanghai enzyme research Biotechnology Co., Ltd. The incidence of adverse anesthetic reactions, including subcutaneous hematoma, pneumothorax, nausea and respiratory depression.

### Statistical analysis:

SPSS 22.0 software was used to process and analyze the collected data. The measurement data were expressed in (*χ̄*±*S*), and t-test was used for comparison between groups; the counting data was expressed in n (%) and was processed by *χ^2^* test. *P*<0.05 indicated statistical significance.

## RESULTS

There was no significant difference in gender, age, body mass index (BMI), American Society of Anesthesiologists (ASA) grade, operation time and blood loss between the two groups (*P*>0.05) Table-I. There was no significant difference between the onset time of block and anesthesia plane between the two groups (*P*>0.05), but the duration of block in SAPB-group was higher than that in TPVB-group (*P*<0.05) Table-II.

**Table-I:** Comparison of general data and perioperative indexes between the two groups (*χ̅*±*S*).

Group	n	Gender		Age (years)	BMI (kg/m^2^)	ASA(n)		Operation time (minute)	Surgical blood loss (mL)
	
		M	F			I	II		
SAPB-group	59	37	22	52.01±11.41	22.45±2.70	44	15	140.23±28.64	89.67±10.38
TPVB-group	55	29	26	53.03±10.76	22.58±2.51	40	15	137.14±28.61	90.49±9.98
*χ*^2^/t		1.164		0.490	0.275	0.050		0.576	0.426
*P*		0.281		0.625	0.784	0.823		0.566	0.671

**Table-II:** Comparison of anesthetic effects between the two groups (*χ̅*±*S*).

Group	n	Block onset time (minute)	Duration of block (hours)	Anesthesia plane (T)
SAPB-group	59	8.59±0.89	11.06±1.71	5.20±1.17
TPVB-group	55	8.71±1.04	9.83±1.30	4.89±1.10
*t*	-	0.637	4.342	1.466
*P*	-	0.526	<0.001	0.146

VAS score of the patients in the SAPB-group was significantly lower than that of TPVB-group at rest and when coughing at all-time points after the surgery (*P*<0.05). The only exception was the VAS score at rest, 48 hours after the operation, which was similar in both groups (*P*>0.05) Table-III.

**Table-III:** Comparison of VAS pain scores at each time point after the operation between the two groups (score,*χ̅*±*S*).

Group	n	At rest					When coughing				

		T1	T2	T3	T4	T5	T1	T2	T3	T4	T5
SAPB-group	59	1.55±0.65	1.81±0.54	2.44±0.85	2.67±0.87	1.93±0.64	2.37±0.64	2.71±0.78	3.18±1.28	3.67±1.38	2.62±0.66
TPVB-group	55	2.01±0.68	2.41±0.68	2.81±1.00	3.12±1.08	1.98±0.68	2.65±0.77	3.07±0.99	3.74±1.25	4.30±1.18	3.12±1.00
*t*	-	3.682	5.204	2.167	2.412	0.401	2.107	2.150	2.357	2.609	3.116
*P*	-	<0.001	<0.001	0.032	0.017	0.489	0.036	0.034	0.020	0.010	0.002

The levels of IL-6, IL-10 and TNF-α at 48 hours after the operation in both groups were higher than those at the end of the operation (*P*<0.05). SABP was associated with significantly lower levels of IL-6 and TNF-α and significantly higher levels of IL-10 48 hours after the operation as compared to TPVB-group (*P*<0.05) Table-IV. The incidence of postoperative subcutaneous hematoma, pneumothorax, nausea and respiratory depression in the SAPB-group was 5.08%, and there was no significant difference compared to 3.64% in the TPVB-group (*P*>0.05) Table-V.

**Table-IV:** Comparison of T0 and T4 inflammatory levels between the two groups (*χ̅*±*S*).

Groupss	n	IL-6 (ng/L)		IL-10 (ng/L)		TNF-α (ng/mL)	
		
		T0	T5	T0	T5	T0	T5
SAPB-group	59	77.49±8.46	112.86±10.63*	42.32±4.67	87.49±7.70*	12.37±1.73	19.83±2.26*
TPVB-group	55	79.96±8.15	137.87±11.21*	42.58±4.89	74.85±8.23*	12.54±1.64	25.98±2.54*
t	-	1.586	12.218	0.290	8.467	0.545	13.652
P	-	0.116	<0.001	0.773	<0.001	0.587	<0.001

***Note:*** Compared with T0,

* indicates P < 0.05.

**Table-V:** Comparison of postoperative adverse anesthetic reactions between the two groups[n(%)].

Group	n	Subcutaneous hematoma	Pneumothorax	Nausea	Respiratory depression	Total
SAPB-group	59	1 (1.69)	1 (1.69)	1 (1.69)	0	3 (5.08)
TPVB-Group	55	1 (1.82)	1 (1.82)	0	s0	2 (3.64)
*χ* ^2^						0.142
*P*						0.706

## DISCUSSION

The results of this study show that while both ultrasound-guided SAPB block and TPVB block can reduce the pain and control the inflammation in patients undergoing upper abdominal surgery, the effect of SAPB block is significantly better than TPVB block. SAPB block belongs to the category of chest wall blocks. It was first proposed by Blanco et al. in 2013 by improving PEC I and PEC II pathways.^12^ The main mechanism of this procedure is to block the lateral cutaneous branch of intercostal nerve by injecting anesthetic drugs into the space between anterior serratus muscle and latissimus dorsi muscle.^13^

In recent years, with the advances in ultrasound technology and anesthesiology, the efficiency of SAPB block technology and perioperative anesthesia have been significantly improved, resulting in larger sensory block range. The block plane can reach T7~T12, which leads to significant decrease in the postoperative pain level in patients undergoing upper abdominal surgery and is beneficial to patients’ postoperative recovery.^14^

This study found that there was little difference between the onset time of block and anesthesia plane in patients undergoing upper abdominal surgery, but the duration of block in the SAPB-group was longer. This may be related to the anatomical position of the anterior serratus muscle. The SAPB block method belongs to the category of interfacial injection techniques, and targets the lateral cutaneous branches and T2-T6 intercostal nerve.

While TPVB acts directly on the spinal nerves, a single injection may only results in a block range of 2~4 ganglion segments and may not be enough to produce sufficient analgesia. In a randomized controlled trial of 40 cases of radical mastectomy.^15^ Suman Arora showed that the duration of SAPB guided block was significantly longer than that of TPVB (*P*<0.001), which is consistent with the results of this study.^16^ Kulhari et al. found that the pain level of patients after ultrasound-guided thoracic nerve (PECS II) block was significantly better than that of patients with thoracic paravertebral block (TPVB).^17^ The reason may be related to the pharmacological effect of the anesthesia method of thoracic nerve block.

The thoracic nerve block method has a good block effect on the long thoracic nerve and the lateral intercostal nerve. It can significantly reduce the incision pain stimulation caused by intraoperative manipulation and weaken its conduction function to spinal cord sedation.^18^,^19^ The results of our study are in agreement with these observations. While we did not detect significant difference in the VAS score between the two groups at 48 hours after the operation, the VAS score of SAPB-group was significantly lower than that of TPVB-group at all other time points after the operation, whether at rest or when coughing (*P*<0.05).

The results showed that the level of inflammation control in SAPB-group was significantly better than that of TPVB-group. SAPB was associated with significantly lower levels of IL-6 and TNF- α and increased levels of anti-inflammatory cytokine IL-10. As an external stimulus, surgery and surgery-associated pain triggers inflammatory reaction in patients and leads to secretion of a large number of inflammatory factors.^20^ This in turn further aggravates the pain, creating a vicious circle. The use of ultrasound-guided low SAPB block is associated, therefore, with reduced secretion of inflammatory factors and inflammatory response.

In this study, the rate of adverse reactions, such as subcutaneous hematoma, pneumothorax, nausea and respiratory depression, in SAPB-group was 5.08%, similar to 3.64% in TPVB-group, indicating that both methods were equally safe. Previous studies have found that the total incidence of postoperative adverse reactions in SAPB-group was significantly lower than that in TPVB-group, which is different from the conclusions of our study.^21^,^22^ The reason may be related to the sample size included in this study.

### Limitation of the study:

Since it was a retrospective single center study with a small sample size, the generalizability of the results may be limited, and future prospective randomized studies are required to confirm the findings of our study. Only the relevant indexes within 48 hours after the operation were collected. Further multi-center studies with larger cohorts and longer follow-up are merited.

## CONCLUSION

In patients undergoing upper abdominal surgery both SAPB and TPVB have good anesthetic and analgesic effect and can significantly improve the level of postoperative inflammation. We show that SAPB block results in longer duration of the block, significantly lower postoperative VAT scores and reduced inflammatory response as compared to the TPVB block. This study can provide further reference for clinicians.

### Authors’ contributions:

**ZC** conceived and designed the study.

**RX, TX** and **CH** collected the data and performed the analysis.

**ZC** was involved in the writing of the manuscript and is responsible for the integrity of the study.

All authors have read and approved the final manuscript.
